# A Capacitive Particle-Analyzing Smoke Detector for Very Early Fire Detection

**DOI:** 10.3390/s24051692

**Published:** 2024-03-06

**Authors:** Boqiang Wang, Xuezeng Zhao, Yiyong Zhang, Zigang Song, Zhuogang Wang

**Affiliations:** 1 School of Mechatronics Engineering, Harbin Institute of Technology, Harbin 150006, China; zhaoxz@hit.edu.cn; 2 703 Research Institute, China State Shipbuilding Corporation Limited, Harbin 150010, China; 17601527196@163.com (Y.Z.); songasd1981@163.com (Z.S.); 15124588116@163.com (Z.W.)

**Keywords:** extreme early fire detection, smoke concentration detection, capacitive detection, multiscale signal processing

## Abstract

Smoke detectors face the challenges of increasing accuracy, sensitivity, and high reliability in complex use environments to ensure the timeliness, accuracy, and reliability of very early fire detection. The improvement in and innovation of the principle and algorithm of smoke particle concentration detection provide an opportunity for the performance improvement in the detector. This study is a new refinement of the smoke concentration detection principle based on capacitive detection of cell structures, and detection signals are processed by a multiscale smoke particle concentration detection algorithm to calculate particle concentration. Through experiments, it is found that the detector provides effective detection of smoke particle concentrations ranging from 0 to 10% obs/m; moreover, the detector can detect smoke particles at parts per million (PPM) concentration levels (at 2 and 5 PPM), and the accuracy of the detector can reach at least the 0.5 PPM level. Furthermore, the detector can detect smoke particle concentrations at better than 1 PPM accuracy even in an environment with 6% obs/m oil gas particles, 7% obs/m large dust interference particles, or 8% obs/m small dust interference particles.

## 1. Introduction

Very low concentrations of smoke particles can be effectively detected during very early fire detection. This approach can effectively warn of, and thus prevent, the further development of fires and minimize losses of all kinds. Unfortunately, there are more than 100,000 cases of no alarm generation or alarm failure [1], and more than 200,000 false alarms were responded to by fire departments, with these statistics being from the China Emergency Management Department in 2023 [2]. These factors result in unnecessary losses, waste of firefighting resources, and declining public confidence. The fast and accurate detection of smoke particles from fast-spreading fires is critical for avoiding losses and saving lives.

Smoke concentration detection technology confronts the challenges of interfering particles in complex environments, false alarm resistance, and adaptation. Conventional point smoke detectors cannot cope with harsh and intrusive environments [3]. Photoelectric smoke detectors are not in a position to distinguish between particle signals of different sizes, but the detector response speed increases when the emitting light source is a green LED [4]. Very low-concentration smoke particles released from very early fires can be effectively recognized by a photoelectric aspirating smoke detector, and this type of detector has achieved successful commercial application [5]. However, this approach can only partially eliminate the effect of other interfering particles through the filter and cannot distinguish the particle type. These factors significantly limit the applicability of the detector. The impact of the airflow direction on the mounting angle of the detector needs to be considered when designing the layout style of the pipeline [6]; the air sample pipeline needs to be complexly modeled in 3D to verify the reasonableness of the pipeline layout [7]; and the trajectories of smoke particles need to be identified by using computational fluid dynamics [8]. The false alarm resistance of a detector can be improved by adding a combustible gas detection module for alarm calibration [9]. However, this approach also influences the sensitivity of the detector to a certain extent. A capacitive bending smoke sensor can increase its sensitivity by increasing the component contract area. A capacitive smoke sensor based on MEMS technology can detect smoke generated by hydrogen-containing substances during the smoldering stage. However, it is not sensitive to carbon-containing substances and still cannot distinguish the type of smoke particle [10]. Smoke particles can be detected in vacuum environments by utilizing finely machined capacitive sensors, but they still cannot distinguish between particle types [11]. The use of series capacitors can increase the sensitivity of the sensor to smoke particles. However, it is not possible to realize the detection of smoke particles at the PPM level or to distinguish the type of smoke particle [12]. While very low concentrations of smoke particles generated by very early fires are effectively detected, the effective identification of particle types is still a problem. Moreover, the false alarm rate of the detector tends to increase, and its reliability will be greatly affected in complex environments where oil gas particles and dust particles of different sizes are present.

In this study, a structure for analyzing and detecting smoke particles based on capacitive detection element cells is designed, which uses particles of different sizes to form mixed signals with different amplitudes and frequencies when they pass through the detection structure. A multiscale algorithm is used to detect smoke particle concentrations by sequentially analyzing mixed signals via time-frequency domain analysis, extracting smoke particle signals, sensitizing smoke signals, and calculating smoke concentrations. On the one hand, the detector will have higher detection accuracy and sensitivity because smoke particles are identified by the newly designed capacitive detection cell. On the other hand, the detector can differentiate signal characteristics effectively between dissimilar particles through the newly designed particle detection structure and algorithm so that the reliability of the detector increases in complex environments. The sensitivity, accuracy, and reliability of the proposed method were verified through a limit concentration detection experiment, smoke concentration detection experiment, and anti-interference ability experiment, respectively.

## 2. Capacitive Smoke Particle Detection Principle and Design

### 2.1. Capacitive Particle-Analyzing Detector Structure

**Figure 1:**
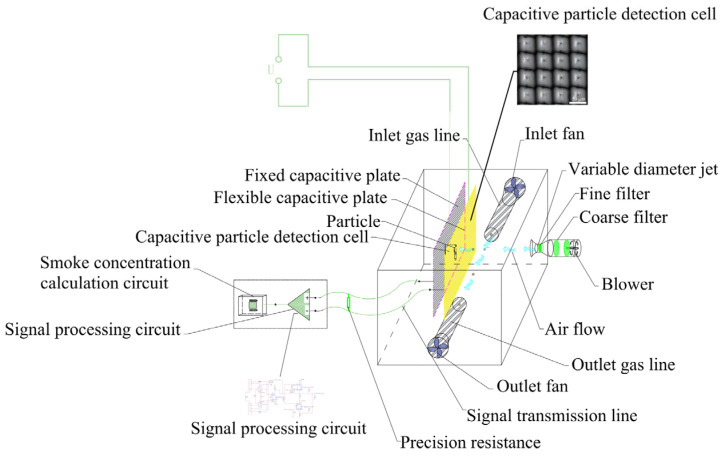
Capacitive particle analysis structure schematic.

As shown in Figure 1, the capacitive particle analysis structure mainly consists of a pair of capacitive particle detection plates, a gas sample sampling path, a motive air path, and a signal processing circuit. Capacitive particle detection plates consist of a fixed capacitive plate and a flexible capacitive plate for detecting the particle type. The gas sample sampling path consists of inlet/outlet fans, inlet/outlet gas lines, and a particle detection chamber to sample the air samples. The power gas path consists of filters, a blower, and a variable diameter jet exhaust to provide the kinetic energy for the sampled air to collide with the flexible capacitive plate.

In addition, the variable diameter jet will blow on the whole detection chamber by changing the shape of its nozzle according to a pre-set program, and will remove all kinds of particles from the detection chamber with the help of airflow formed by the inlet and outlet fan after sending out an alarm signal from the detector.

The power consumption of the detector is 3.6 W, and the noise produced is 35 dB. It is mainly used in the powerhouse of ships but is also suitable for distributed applications in apartments or small buildings.

### 2.2. Particle Detection Principle

**Figure 2:**
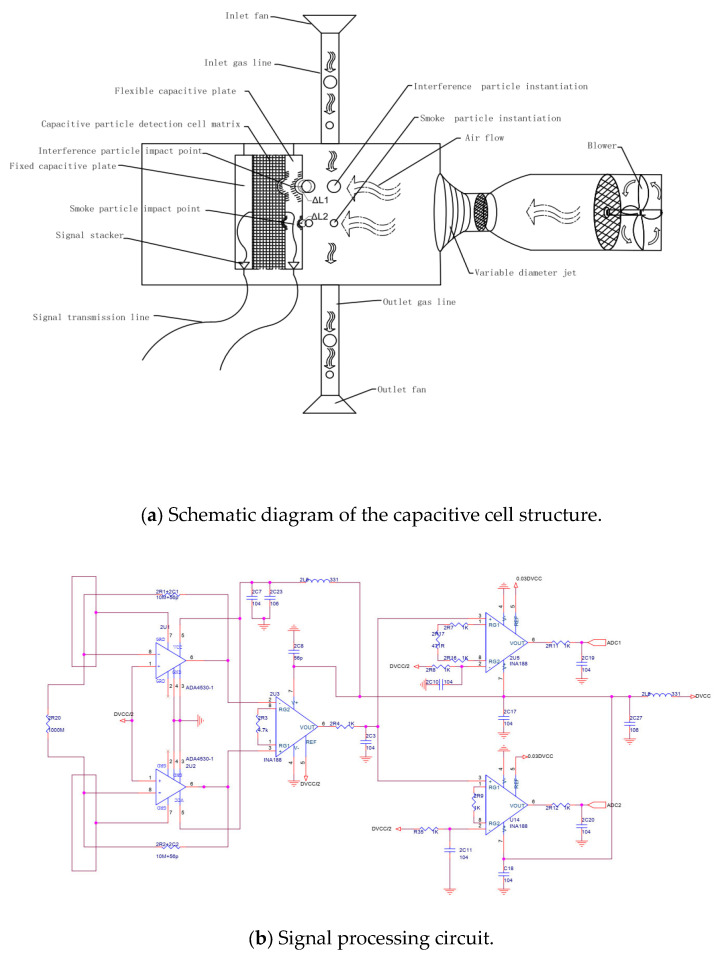
Particle detection schematic and signal processing circuit.

As seen in Figure 2a, smoke particles and interference particles are simultaneously inhaled into the particle detection chamber by the inlet fan. The air inhaled by the blower will be purified into clean power gas after going through two layers of coarse and fine filters. Inhaled smoke particles and interference particles are blown by such gas to the flexible capacitive plate and collide with it. Let us assume that vertical deformations of ${\Delta L}_{1}$ and ${\Delta L}_{2}$ are formed by a collision between interference particles and smoke particles on the flexible capacitive plate, respectively. Then, the capacitance on the capacitance cell changes as follows:

(1) $$\left\{\begin{array}{c} C_{\Delta L_{1}}=\frac{\varepsilon \cdot A}{d-\Delta L_{1}}\\ C_{\Delta L_{2}}=\frac{\varepsilon \cdot A}{d-\Delta L_{2}} \end{array}\right.$$

where $C_{\Delta L_{1}}$ and $C_{\Delta L_{2}}$ are the capacitance variations generated on the impinged capacitance cell by interference particles and smoke particles, respectively; d is the distance between the fixed capacitive plate and the flexible capacitive plate before the collision; ε is the permittivity of the capacitor; and A is the relative projected area of the two capacitive plates. Since the force of the blower does not change during the detection process, and the deformation of the flexible capacitor pole by the blower only occurs when the detector is turned on and the deformation is fixed, the capacitance variation of the capacitor will not be changed by the force of the blower during the detection process.

A fixed DC voltage U is applied between the fixed capacitive plate and the flexible capacitive plate. A precision sampling resistor is connected in series between two signal stackers of the fixed capacitive plate and flexible capacitive plate, and the signal stacker is used to collect the electrical signal produced by capacitive cells. Induced currents flow through the sampling resistor, and a voltage is produced when the change in capacitance is caused by particle impacts on the flexible plate. The fixed DC voltage U is 5 V, the relative projected area of the two capacitive plates A is $25\times 15\;{\mathrm{cm}}^{2}$, and the distance d is 5.6 mm. As shown in Figure 2b, the precision resistor 2R20 (in Figure 2b) is a 1000 MΩ resistor. Precision resistors only mean the precision of the resistance value, which here is 0.01%. The signal stackers are the operational amplifiers 2U1 and 2U2 in Figure 2b and are used to sample the voltage across the precision resistor 2R20.

### 2.3. Capacitor Detection Cell Design

Owing to the blower, the capacitive cell’s vertical orientation detection capability is applicable only since particles collide with the flexible plate in that orientation, and the cutting orientation detection capability of the capacitive cell does not have to be considered.

**Figure 3:**
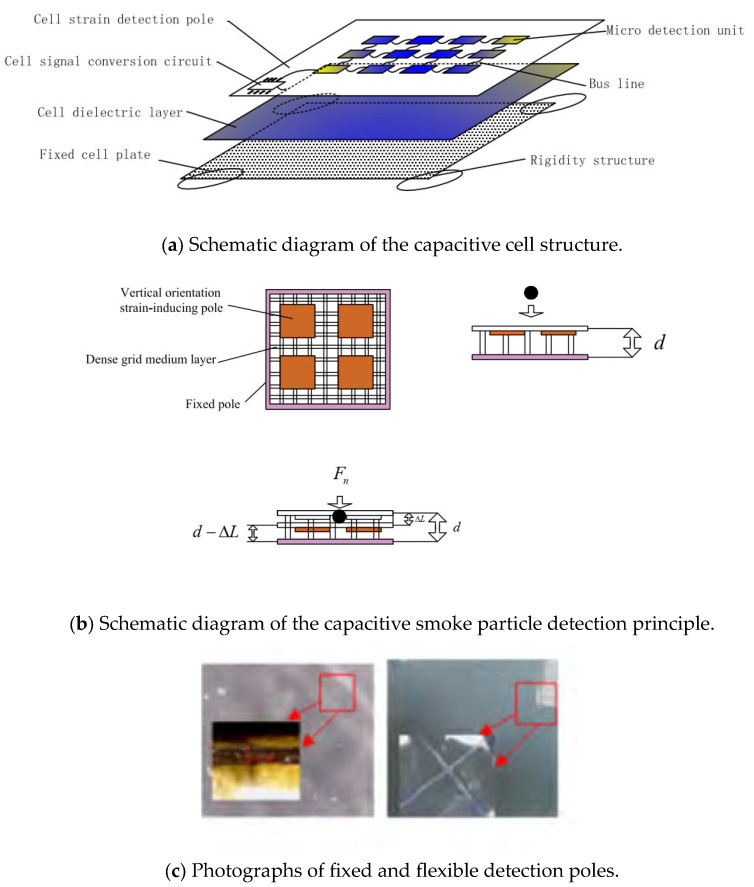

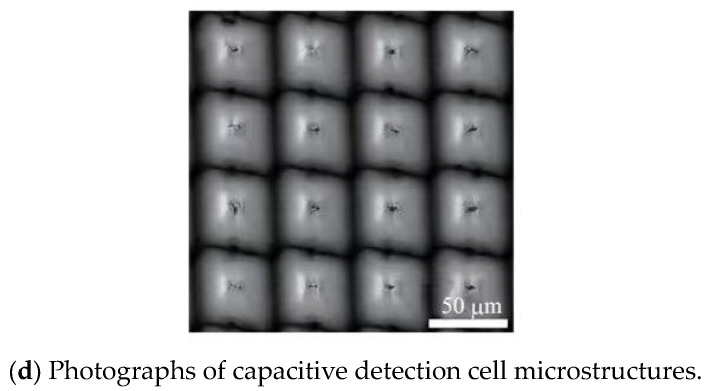
Capacitive detection cell.

The perpendicular orientation of the capacitive cell is designed based on the dense grid medium, as shown in Figure 3a. It mainly consists of a cell strain detection pole, a cell dielectric layer, and the fixed-cell plate, and the cell strain detection pole consists of several microdetection units connected by a bus line. Eventually, the electrical signal from the microdetection units is collected and converted by the cell signal conversion circuit. The cell dielectric layer is composed of a frothy silicon–lipid mixture. The fixed-cell plate design is built on rigid structures that prevent cutting orientation movement from affecting cell detection accuracy.

As seen in Figure 3b, the smoke particles collide with the vertically oriented strain-inducing pole of the capacitive cell under the action of the blower. Under the effect of the collision force $F_{n}$, the dense grid medium will be compressed, which will change the distance between the fixed plate and the strain-inducing pole, thus changing the capacitance value of the capacitor. The detection of smoke particles is obtained by detecting the change in electrical signals caused by changes in capacitance. The capacitive detector cell is designed with a micro-nano structure, making it sensitive enough to detect smoke particles at PPM-level concentrations. The size of the capacitive detection cell is 45 μm. The material of the detection cell is carbon fiber.

Assuming that the invariant of the cell microdetection unit is ΔL after collision with particles, it can be expressed as follows [13]:

(2) $$\Delta L=\frac{F_{n}d}{\rho_{A}EA_{S}}$$

where $\rho_{A}$ is the filling rate of the cell dielectric layer, E is the elastic recovery of the cell dielectric layer, and $A_{S}$ is the area of the cell dielectric layer.

$F_{n}$ can be expressed as follows [14]:

(3) $$F_{n}=\delta_{i}F_{fan}R_{i}$$

where $\delta_{i}$ is the inertia coefficient of particle type i, $R_{i}$ is the diameter of particle type i, and $F_{fan}$ is the driving force of the blower to the particles. Furthermore, capacitance variations can be obtained after the cell microdetection unit collides with particles, as shown in Equation (4), and the sensitivity can be expressed by Equation (5).

(4) $$C_{\Delta L_{i}}=\frac{\varepsilon A_{i}}{d-\Delta L_{i}}=\frac{\varepsilon A_{i}}{d-\frac{\delta_{i}F_{fan}R_{i}d}{\rho_{A}EA_{S}}}$$

(5) $$\frac{\partial C_{\Delta L_{i}}}{\partial R_{i}}=\frac{\varepsilon A_{i}\rho_{A}EA_{s}}{d{\left(\rho_{A}EA_{S}-\delta_{i}F_{fan}R_{i}d\right)}^{2}}$$

where $A_{i}$ is the sensing electrode area of the microdetection unit and $\Delta L_{i}$ is the invariant of the cell microdetection unit after collision with particles. Because $F_{n}$ has a much smaller impact than $\rho_{A}EA_{s}$, the impact $F_{n}$ can be ignored. At this point, the sensitivity can be expressed as follows:

(6) $$\frac{\partial C_{\Delta L_{i}}}{\partial R_{i}}=\frac{\varepsilon A_{i}}{d\rho_{A}EA_{s}}$$

A mixture of flexible body and gas gaps form between the cell strain detection pole and the fixed cell plate. Equation (6) shows that the filling rate of the mixture on the cell dielectric layer should be reduced to improve the sensitivity. The minimum particle diameter that can be detected by the detector is 0.5 μm, its mass is 0.16 μg, and the detector’s sensitivity is 5.14 μf/μm.

## 3. Signal Output Model and Algorithm Model

### 3.1. Model of the Output Signal from the Particle Analysis Structure

The capacitance changes when the flexible capacitive plate collides with particles. Because a fixed DC voltage is applied between two plates, an alternating current will produce a change in capacitance, the amplitude of which is the superposition of all weak AC signals caused by collisions between particles (including smoke particles and interfering particles) and capacitive cells, and the signal will be output by the signal stacker between two plates [15].

(7) $$I_{sum}=U\cdot \frac{dC_{sum}}{dt}=U\cdot [\frac{dC_{\Delta L_{1}}}{dt}+\frac{dC_{\Delta L_{2}}}{dt}+\ldots +\frac{dC_{\Delta L_{i}}}{dt}]$$

where $I_{sum}$ is the total alternating current signal synthesized by the signal stacker and $C_{sum}$ is the superposition of changes in the capacitance of the capacitor. The AC voltage signal is obtained on the precision resistor in series between two signal stacks [16].

(8) $$U_{sum}=I_{sum}*R_{samp}$$

where $R_{samp}$ is the electrical resistance of the precision sampling resistor and $U_{sum}$ is the AC voltage applied to the precision sampling resistor. A superposition of sinusoidal voltages with different frequencies and amplitudes will be formed after filtering and amplification by the signal processing circuit (as illustrated in Figure 1) [17].

(9) $$U\left(t\right)=\sum_{R_{i}=R_{s},R_{N_{1}},R_{N_{2}}\cdots }A_{R_{i}}\cdot \sin\left[\omega_{R_{i}}\cdot t+\varphi \right]$$

where $R_{i}$ is the diameter of different particles, $R_{s}$ is the diameter of smoke particles to be detected, $R_{N_{1}}$, $R_{N_{2}}$, etc. are the diameters of interfering particles, $\omega_{R_{i}}$ is the frequency of the signal produced by particles with a diameter $R_{i}$, $A_{R_{i}}$ is the amplitude of the signal produced by particles with a diameter $R_{i}$, φ is the offset angle of the signal, and t is the time.

### 3.2. Smoke Concentration Detection Algorithm

#### 3.2.1. Overall Design of the Multiscale Smoke Particle Concentration Detection Algorithm

**Figure 4:**
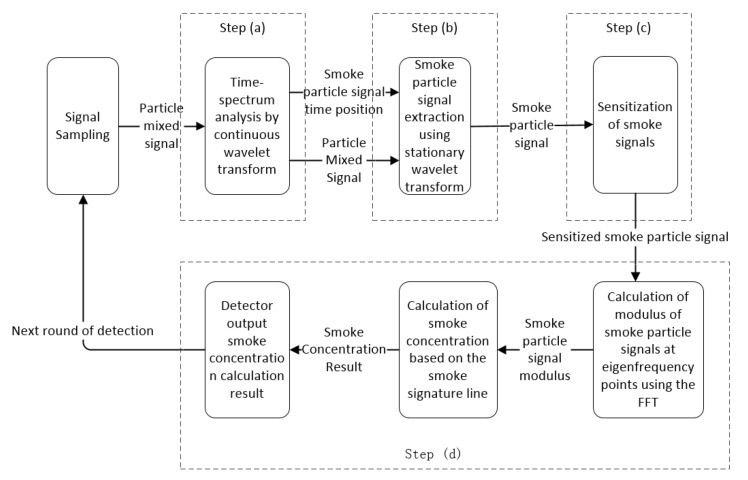
A flowchart of the multiscale smoke particle concentration detection algorithm.

The signal output of the detector is formed in part by the superposition of signals generated by particles at different times. The weak signal needs to be amplified with the signal enhancement technique because the size of the smoke particles is insignificant, as shown in Figure 4. These drawbacks stop the use of a single method for signal processing from meeting the demand for smoke concentration detection. The multiscale smoke concentration detection algorithm is a combinatorial algorithm of a continuous wavelet transform, a smooth wavelet transform, the sensitization of smoke signals, and single-frequency point concentration calculations. Therefore, the multiscale smoke concentration detection algorithm—a combination of multiple signal analysis methods—will be used for this detection, and its main steps can be divided as follows:

(a) First, the time position of the smoke particle signal in the detector output signal is determined.
(b) After that, the smoke particle signal needs to be extracted.
(c) Subsequently, the signal after extraction is sensitized and amplified.
(d) Finally, the smoke concentration is calculated via single-frequency analysis.

#### 3.2.2. Time–Frequency Analysis of Signals

First, a time–spectrum analysis of the detector output signal is performed by using a continuous wavelet transform along the time axis, and the moment at which the smoke particle signal appears is determined. The continuous wavelet transform of the continuous signal $f\left(t\right)$ can be expressed as follows [16]:

(10) $$WT_{f}\left(a,b\right)=\langle f(t),\psi_{a,b}\left(t\right)\rangle \frac{1}{\sqrt{a}}\int_{-\infty }^{+\infty }f\left(t\right)\psi^{*}\left(\frac{t-b}{a}\right)dt$$

where a is the scale parameter of the wavelet function, b is the translation parameter of the wavelet function, $\psi_{a,b}\left(t\right)$ is the wavelet basis function for parameters a and b, $\psi^{*}\left(t\right)$ is the conjugate function of the wavelet basis function, and $f\left(t\right)$ is the source signal function.

The relationship between the wavelet decomposition scale and signal frequency after transformation can be expressed as follows [18]:

(11) $$f_{a}=\frac{f_{c}f_{s}}{a}$$

where $f_{a}$ is the actual signal frequency after decomposition, $f_{c}$ is the center frequency of the wavelet basis function, and $f_{s}$ is the sampling frequency of the signal. According to the sampling theorem, the value ranges of the scale parameter are satisfied $a\in \left[2f_{s},\infty \right]$ so that the value ranges of the frequency of the wavelet basis function can be satisfied $f_{c}\in \left[0,f_{s}/2\right]$.

#### 3.2.3. Smoke Particle Signal Separation

In addition, the smoke particle signal is extracted from the detector output signal by a stationary wavelet transform.

In the stationary wavelet transform, the scale parameter a needs to be discretized, and the translation parameter b must remain constant so that the signal after the transform has the same length as the original signal $f\left(t\right)$. The stationary wavelet transform can be obtained through discrete sampling of the scale parameter a within the binary sequence $\left\{2^{j}\right\}$ (where j∈Z) [19].

(12) $$SWT_{f}\left(j,b\right)=\langle f\left(t\right),\psi_{a,b}\left(t\right)\rangle =\frac{1}{\sqrt{2^{j}}}\int_{-\infty }^{+\infty }f\left(t\right)\psi^{*}\left(\frac{t-b}{2^{j}}\right)dt,j\in Z$$

Equation (12) shows that only the scale parameter a is discretized by the stationary wavelet transform, and the translation parameter b remains constant. In this way, the wavelet coefficients are all retained, and the length of the wavelet coefficients remains constant after each transform.

There are two ways of upsampling and downsampling at the same time so that the lengths of the signal between the original signal and the high- and low-frequency coefficients after the transform remain constant when the original signal is disintegrated by the stationary wavelet transform. This sampling mode is achieved by interpolating $2^{j}$ zeros between the two coefficients of the high-pass and low-pass filters; the high-pass and low-pass filter coefficients are stripped in this way. The high-pass and low-pass filters in the transformation can be expressed as follows [20]:

(13) $$g\left(k\right)=\left\{\begin{array}{c} g\left(\frac{k}{2^{j}}\right),\;k=2^{j}m\\ 0,\;others \end{array}\right.$$

(14) $$h\left(k\right)=\left\{\begin{array}{c} h\left(\frac{k}{2^{j}}\right),\;k=2^{j}m\\ 0,\;others \end{array}\right.$$

where j,k,m∈Z, $g\left(k\right)$ and $h\left(k\right)$ denote the unit response functions of the high-pass and low-pass filters, respectively.

Furthermore, the decomposition based on the Mallat algorithm can be obtained as follows [21]:

(15) $$\left\{\begin{array}{c} S_{j+1}\left(n\right)=\sum_{k=1}^{M}S_{j}\left(k\right)g^{*}\left(k-2n\right)\\ d_{j+1}\left(n\right)=\sum_{k=1}^{M}d_{j}\left(k\right)h^{*}\left(k-2n\right) \end{array},\;j=0,1,\cdots J\right.$$

where j is the decomposition depth of the Mallat algorithm, J is the number of decompositions of the signal, n is the degree of decomposition of the signal, k is the order number of the decomposed sequence, M is the sampling point upper limit of the decomposed sequence, and $S_{j}\left(k\right)$ and $d_{j}\left(k\right)$ denote the coefficients of the high-pass and low-pass filters, respectively, at the *j*th signal decomposition.

The detector output signal, which includes the smoke particle signal period, is decomposed by the stationary wavelet transform based on the Mallat algorithm. Let us assume that the eigenfrequency of the awaiting detection smoke particle signal is $\omega_{R_{S}}$ and that the eigenfrequency of the interfering particle signal is $\omega_{R_{i}}$. The signal that contains only smoke particles can be acquired after the i step of stationary wavelet decomposition.

**Figure 5:**
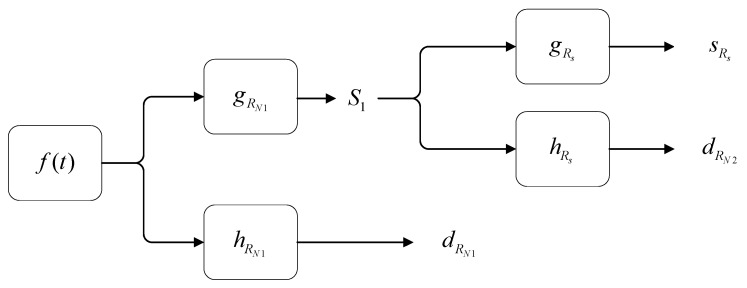
Signal decomposition diagram of the detector output signal obtained by the stationary wavelet transform.

In Figure 5, 2-s2-step decomposition is shown as an example. First, the original signal $f\left(t\right)$ is decomposed by high-pass and low-pass filters with coefficients $g_{R_{N_{1}}}$ and $h_{R_{N1}}$, respectively, and the signal $S_{1}$ filters the interference caused by interference particles of size $R_{N_{1}}$ and the interference signal $d_{R_{N_{1}}}$ generated by particles of this size. Subsequently, the signal $S_{1}$ is decomposed again by another high-pass and low-pass filter with coefficients $g_{R_{S}}$ and $h_{R_{S}}$, respectively, and the signal $S_{R_{S}}$ contains only the signal generated by smoke particles and the signal $d_{R_{N_{2}}}$ generated by interference particles of size $R_{N_{1}}$.

The relationship between the coefficients $g_{R_{N_{1}}}$ and $h_{R_{N_{1}}}$ of high-pass and low-pass filters in the first decomposition layer and the eigenfrequency $\omega_{R_{N_{1}}}$ of the interference signal caused by particles with size $R_{N_{1}}$ can be expressed as follows [22]:

(16) $$g_{R_{N_{1}}}=\beta_{R_{N_{1}}}\omega_{R_{N_{1}}}g\left(\frac{k_{N_{1}}}{2^{j_{N_{1}}}}\right)$$

(17) $$h_{R_{N_{1}}}=\beta_{R_{N_{1}}}\omega_{R_{N_{1}}}h\left(\frac{k_{N_{1}}}{2^{j_{N_{1}}}}\right)$$

where $g\left(\frac{k_{N_{1}}}{2^{j_{N_{1}}}}\right)$ and $h\left(\frac{k_{N_{1}}}{2^{j_{N_{1}}}}\right)$ are the unit response functions of the high-pass and low-pass filter decomposition depths, respectively, and $N_{1}$ and $\beta_{R_{N_{1}}}$ are the correction coefficients for the eigenfrequency $\omega_{R_{N_{1}}}$.

Similarly, the relationship among the coefficients $g_{R_{S}}$ and $h_{R_{S}}$ of the high-pass and low-pass filters in the second decomposition layer and the eigenfrequency $\omega_{R_{s}}$ of the smoke signal caused by particles of size $R_{s}$ can be expressed as follows [23]:

(18) $$g_{R_{S}}=\beta_{R_{S}}\omega_{R_{S}}g\left(\frac{k_{N_{S}}}{2^{j_{N_{S}}}}\right)$$

(19) $$h_{R_{S}}=\beta_{R_{S}}\omega_{R_{S}}h\left(\frac{k_{N_{S}}}{2^{j_{N_{S}}}}\right)$$

where $g\left(\frac{k_{N_{S}}}{2^{j_{N_{S}}}}\right)$ and $h\left(\frac{k_{N_{S}}}{2^{j_{N_{S}}}}\right)$ are the unit response functions of the high-pass and low-pass filter decomposition depths, and $N_{S}$ and $\beta_{R_{S}}$ are the correction coefficients for the eigenfrequency $\omega_{R_{s}}$.

#### 3.2.4. Signal Sensitization and Smoke Concentration Calculation

**Figure 6:**
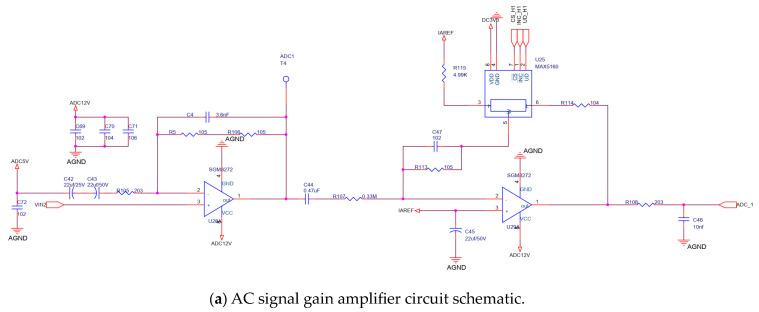

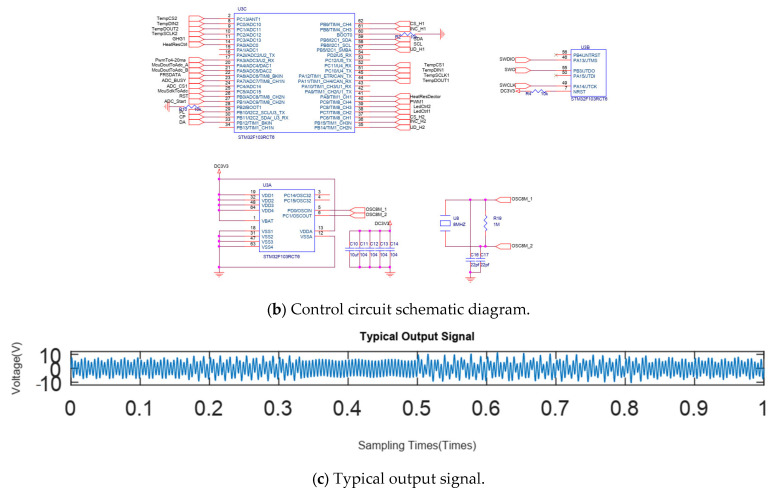
Circuit schematic and typical output signal.

A programmable circuit, as shown in Figure 6, is included in the signal processing circuit in Figure 1. The circuit comprises two operational amplifiers (op. amps.), U28A and U29A, and a digital potentiometer U25. The very low-amplitude raw output at the sensitive element is amplified through a two-stage amplifier circuit comprising U28A and U29A. The gain of the output signal can be adapted by changing the tap position of the digital potentiometer U25. Finally, the processed analog signal is sent to an analog-to-digital converter (ADC).

(20) $$S_{R_{S}}^{*}=S_{R_{S}}\times Gain$$

where $S_{R_{S}}^{*}$ is the sensitized smoke particle concentration signal and Gain is the signal magnification.

The fast Fourier transform (FFT) algorithm was utilized to calculate the modulus of a single frequency point after separation and sensitization. Near the characteristic frequency *ω* of the smoke particle signal, the characteristic frequency modulus $M_{R_{S}}$ can be obtained.

Finally, the smoke concentration can be calculated by bringing the modulus $M_{R_{S}}$ into the smoke concentration characterization line as follows [24]:

(21) $$Col_{R_{S}}=\gamma_{R_{S}}\times M_{R_{S}}+\rho_{R_{S}}$$

where $Col_{R_{S}}$ is the calculated smoke concentration, $\gamma_{R_{S}}$ is the slope of the smoke concentration characteristic line, and $\rho_{R_{S}}$ is the constant of the smoke concentration characteristic line.

## 4. Experimental

### 4.1. Introduction of the Experimental Device

**Figure 7:**
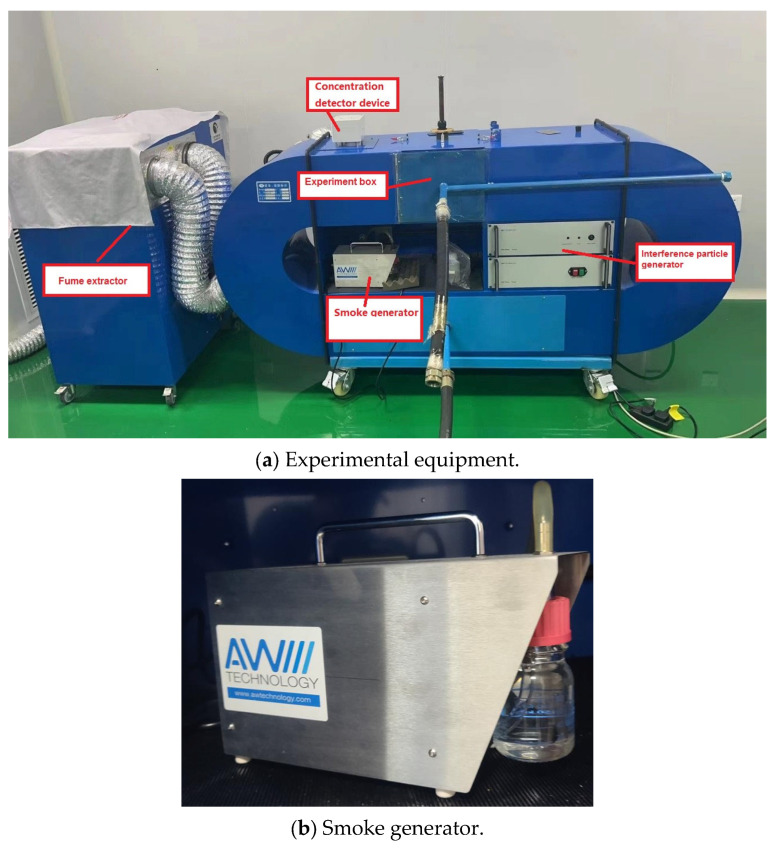

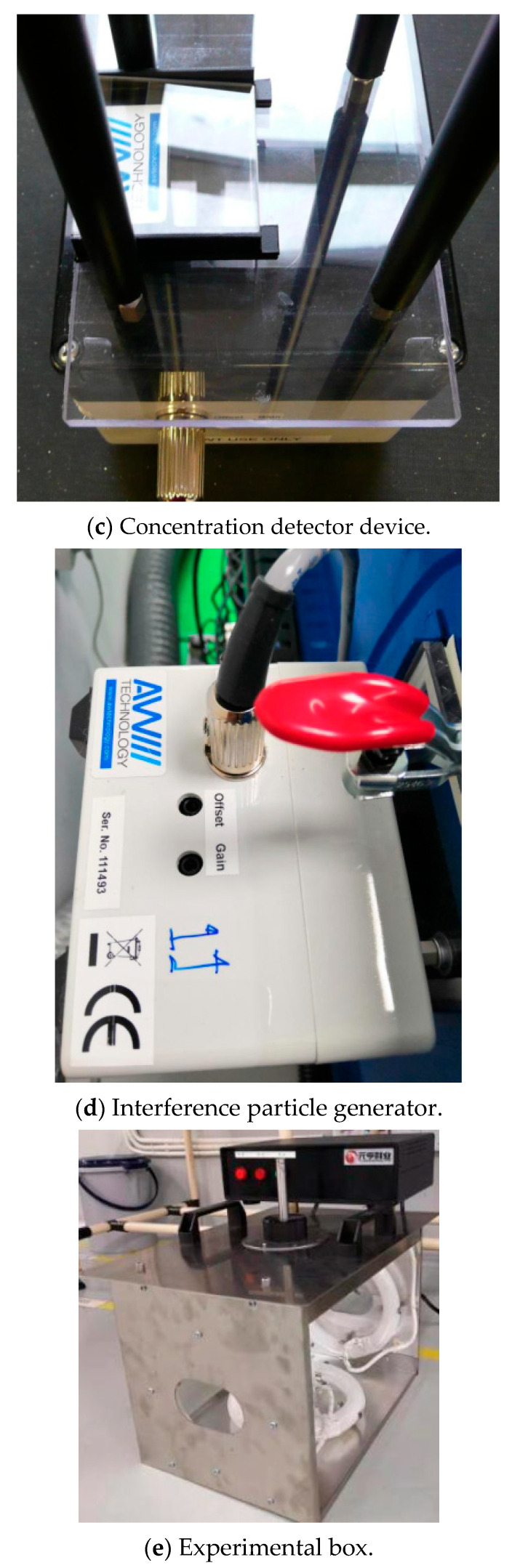
Experimental equipment and part details.

A smoke concentration experimental device was used to test the performance of this detector, as shown in Figure 7. The experiment box is the chamber that holds the detector used in the experiment. The experimental equipment is produced by Beijing Yuanhengliye Corporation (Beijing, China), and its model number is SMK-2000. This experimental device is composed of a smoke particle generator, an interference generator, a concentration detection device, an experiment box, etc. The smoke particle generator generates simulated smoke particles at different concentrations during a fire. An interference generator generates oil gas or dust particles of different sizes and concentrations in different environments. The flue mixture of the above particles was generated, and uniform particles were mixed into the experimental box when the concentration detected by the concentration detection device reached the set conditions. The concentration accuracy of various particles generated by this device (as shown in Figure 7a) is 0.0001 PPM. Particle concentration was measured by an optical densitometer (as shown in Figure 7c). Dust particles are made up of quicklime, while oil gas particles are composed of gasified diesel oil.

### 4.2. Limit of the Concentration Detection Experiment

**Figure 8:**
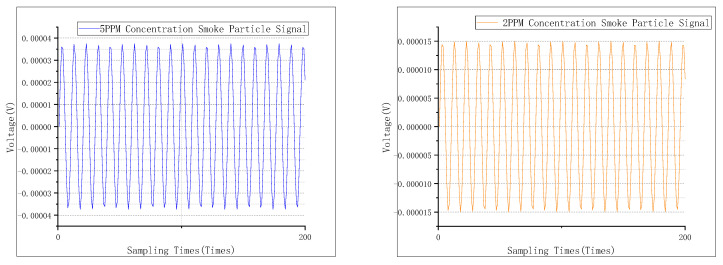
Time domain signal for limit of concentration detection.

**Figure 9:**
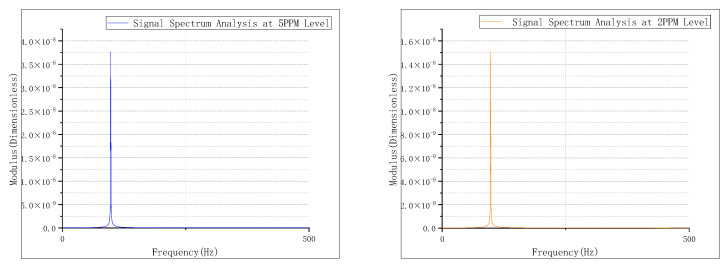
Limit concentration detection spectrum.

The smoke particles were separated at concentrations of 2.0 ppm and 5.0 ppm by this device, after which these particles were used to conduct a concentration limit detection experiment on the detector. The time domain signal of the smoke particle output from the detector is shown in Figure 8, and its spectrum is given in Figure 9. The eigenfrequency $\omega_{R_{s}}$ of the smoke particles can be found to be 210 Hz.

The exact calculations are shown in Table 1, and the deviations are expressed on a parts-per-million (PPM) scale. The deviation is the difference between the concentration (the value shown on the concentration meter on the test set) produced by the device (shown in Figure 7) and the actual concentration (the concentration is calculated by inputting the modulus calculated by the detector at the smoke particle characteristic frequency point $\omega_{R_{s}}$ into Equation (20)) measured by the detector.

As shown in Table 1, the results are 5.2 PPM and 2.3 PPM, with a detection deviation of less than 0.5 PPM when the detector detects smoke particles at concentrations of 2 PPM and 5 PPM, respectively.

### 4.3. Smoke Concentration Detection Experiment

**Figure 10:**
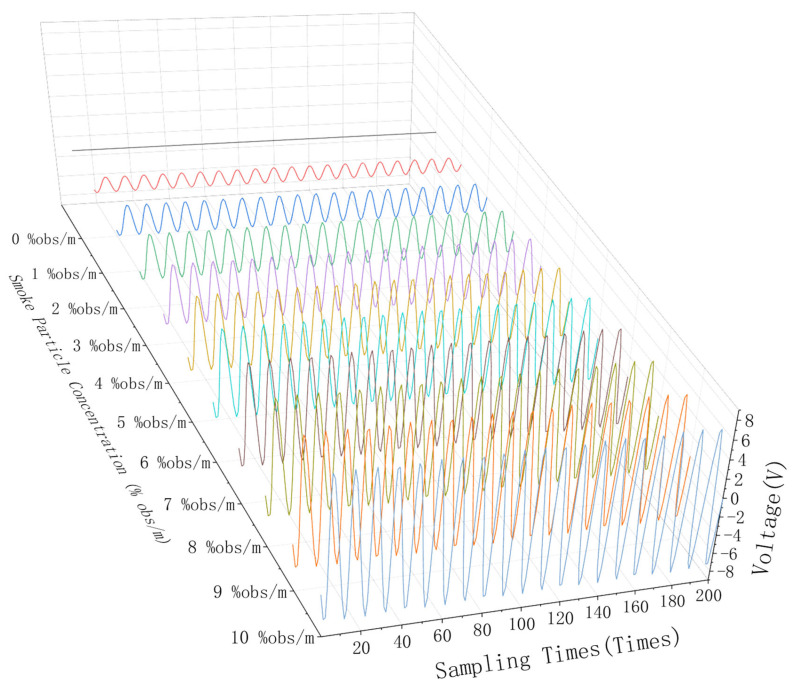
Time domain signal of 0–10% obs/m smoke particle concentration.

**Figure 11:**
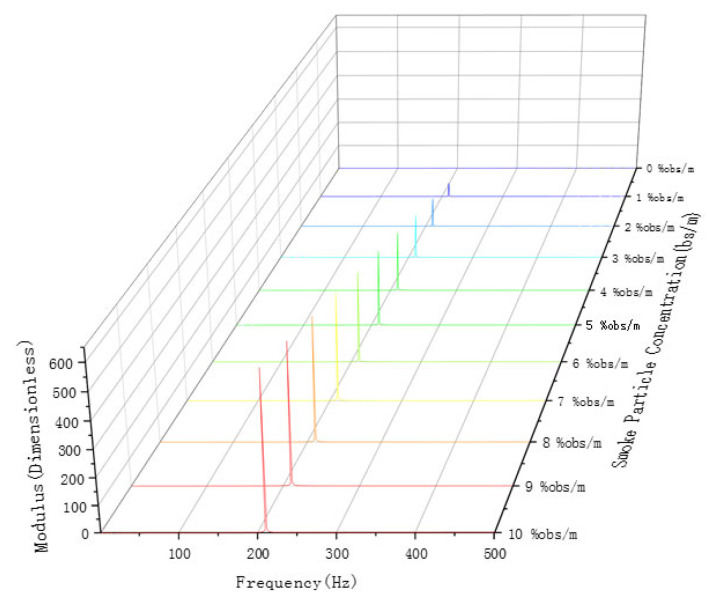
Frequency domain signal of 0–10% obs/m smoke particle concentration.

Smoke particles with concentrations ranging from 0% obs/m to 10% obs/m were separated by this device, and these particles were used to conduct a concentration limit detection experiment on the detector. The time domain and signal spectrum are shown in Figure 10 and Figure 11, respectively, and the detection results are shown in Table 2.

### 4.4. Anti-Interference Ability Experiment

Mixed particles with 6% obs/m oil gas particles, 7% obs/m large dust interference particles, 8% obs/m small dust interference particles, and 2% obs/m smoke particles were prepared, and mixed particles were pumped into the experimental box of this device for an anti-interference experiment.

**Figure 12:**
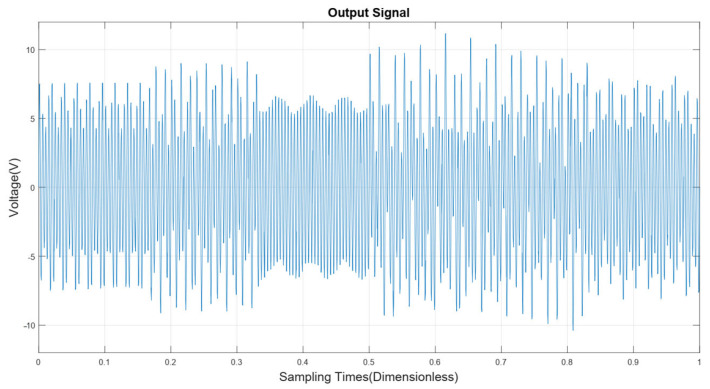
Interference experiment detector signal output diagram.

**Figure 13:**
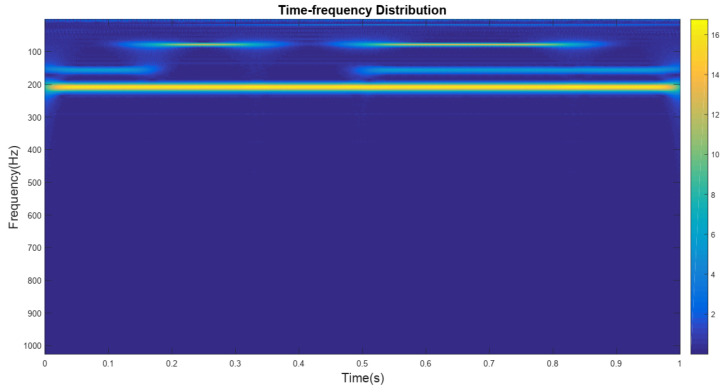
Time–frequency distribution.

The signal output from this detector is shown in Figure 12. Subsequently, the signal of various mixed particles is transformed by a continuous wavelet transform to obtain the time–frequency distribution, as shown in Figure 13. From that figure, it can be seen that there are 4 main frequencies, and the signal with a frequency of 210 Hz is distributed over the whole timeline. This phenomenon occurs because smoke particles, which have a much smaller particle size (usually on the μm level) compared to other interfering particles, are more uniformly distributed in the mixed particles. Therefore, the detector can maintain a uniform number of smoke particles colliding with the detection cell at all times.

**Figure 14:**
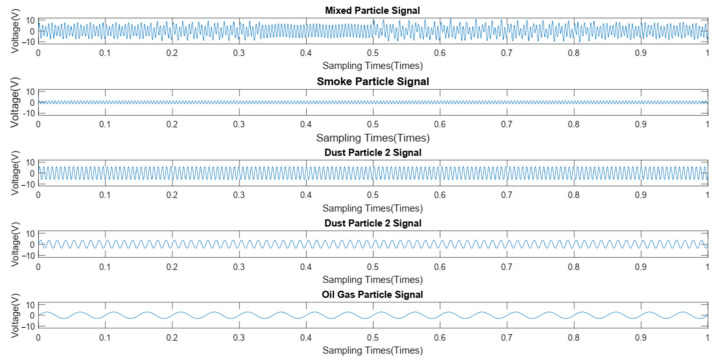
Signal decomposition diagram.

**Figure 15:**
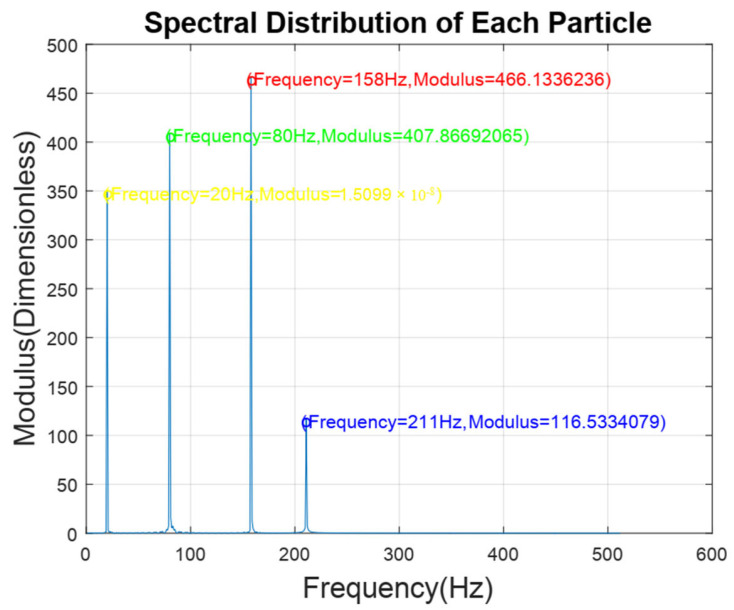
Spectral distribution of each particle.

Furthermore, the signals generated by mixed particles are decomposed to obtain the smoke particle signal. The time domain diagrams before and after signal decomposition are shown in Figure 14. Then, a spectral analysis of the various particle signals after decomposition was performed, as shown in Figure 15. It is apparent from this figure that there are 4 main frequency points at 20 Hz (oil gas particle signal), 80 Hz (large dust interference particle signal), 158 Hz (small dust interference particle signal), and 210 Hz (smoke particle signal). The result is shown in Table 3.

For different concentration combinations of each type of particle, the characteristic frequency of each particle remains constant, and only the modulus changes, because the characteristic frequencies of each type of particle are only related to their size.

### 4.5. Anti-Water Vapor Interference Experiment

**Figure 16:**
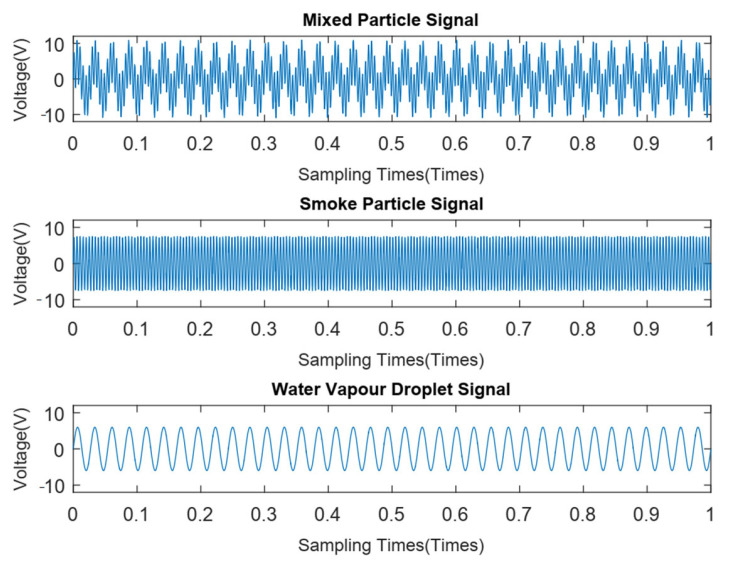
Signal decomposition diagram of false alarm experiment using water vapor.

**Figure 17:**
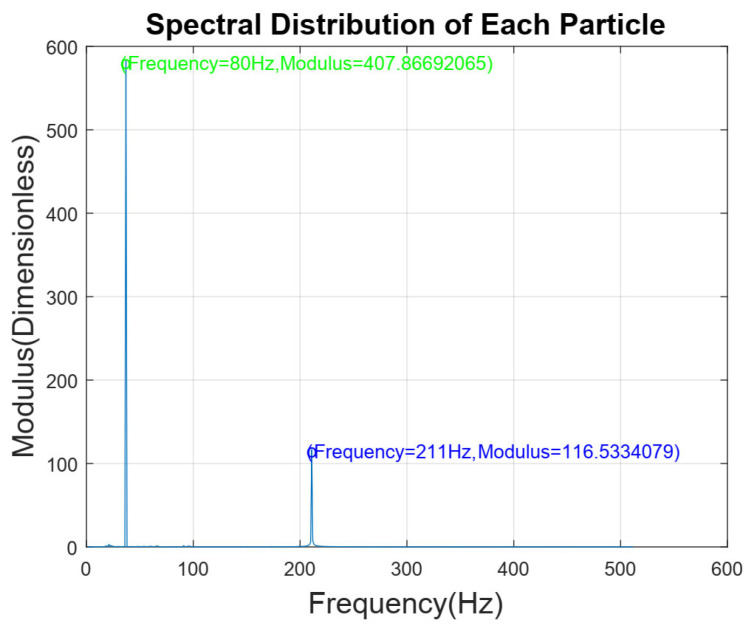
Spectral distribution of each particle.

To verify the anti-false alarm performance of the sensor in humid environments, a certain amount of water vapor was generated by an air humidifier. The sensor then inhaled water vapor and introduced smoke particles at a concentration of 2% obs/m into the detector. The signal is shown in Figure 16. Then, a spectral analysis of 2 particle signals after decomposition was performed, as shown in Figure 17. It is apparent from this figure that there are 2 main frequency points at 37 Hz (water vapor particle signal) and 210 Hz (smoke particle signal).

### 4.6. Anti-High-Density Electrically Conductive Salt Spray Particle Interference Experiment

To verify the anti-false alarm performance of the sensor in marine environments, a certain amount of high-density electrically conductive salt spray particles was generated by an air humidifier by adding ocean saltwater to it. The sensor then inhaled electrically conductive salt spray particles and introduced smoke particles at a concentration of 2% obs/m into the detector. The experiment results are 2 obs/m having just been turned on and also 1 week later, as shown in Table 4.

## 5. Conclusions

(1) When the detector detects smoke particles with concentrations of 2 PPM and 5 PPM, the results were 2.3 PPM and 5.2 PPM, and deviations were less than 0.5 PPM. The following is illustrated by these results: The limit of the smoke particle concentration measured by the detector reaches the PPM level. The designed capacitive detection cell effectively improves the sensitivity of the detector and can measure the concentration of smoke particles effectively at the PPM level.
(2) When the detector detects smoke particles with concentrations of 0–10% obs/m, the resulting deviations were less than 0.5 PPM. The following is illustrated by this result: The designed detector can effectively detect smoke particles at a concentration of 0–10% obs/m, and the detection accuracy can be higher than that of the PPM level. The newly designed capacitive particle analysis detector and multiscale smoke particle concentration detection algorithm can carry out high-precision detection of smoke particles at various concentrations.
(3) When the detector detects the mixed particles (6% obs/m oil gas particles, 7% obs/m large size dust interference particles, 8% obs/m small size dust interference particles, and 2% obs/m smoke particles), the detection result of smoke particles was 2.0000007% obs/m, and the deviation was less than 1 PPM. The following is illustrated by these results: even when there is interference from oil, gas, or dust particles, the detector can still accurately detect at a higher level than the PPM level. This paper shows that capacitive particle analysis and detection structures based on capacitive detection cells combined with a multiscale smoke particle concentration detection algorithm can effectively improve the reliability of detectors to eliminate the influence of other interfering particles on detector performance in complex environments.

## Figures and Tables

**Table 1:** Smoke limit concentration experiment results.

Smoke Concentration (PPM)	Modulus (Dimensionless)	Detection Concentration (PPM)	Deviation (PPM)
2 PPM	0.000150994058	2.3 PPM	0.2 PPM
5 PPM	0.0003774835145	5.2 PPM	0.3 PPM

**Table 2:** Smoke concentration experiment results.

Smoke Concentration (%obs/m)	Modulus (Dimensionless)	Detection Concentration (%obs/m)	Deviation (PPM)
1	58.2667029	1.0000003	0.3 PPM
2	116.5334059	2.0000002	0.2 PPM
3	174.8001084	3.0000003	0.3 PPM
4	233.0668105	4.0000004	0.4 PPM
5	291.3335132	5.0000003	0.3 PPM
6	349.6002158	6.0000002	0.2 PPM
7	407.8669182	7.0000003	0.3 PPM
8	466.1336209	8.0000004	0.4 PPM
9	524.4003241	9.0000002	0.2 PPM
10	582.6670265	10.0000003	0.2 PPM

**Table 3:** Anti-interference ability experiment results.

Smoke Concentration (%obs/m)	Modulus (Dimensionless)	Detection Concentration (%obs/m)	Deviation (PPM)
2	116.5334079	2.0000007	0.7 PPM

**Table 4:** Anti-high-density electrically conductive salt spray particle interference experiment results.

Time	Smoke Concentration (%obs/m)	Modulus (Dimensionless)	Detection Concentration (%obs/m)	Deviation (PPM)
Just turned on	2	116.5334079	2.0000007	0.7 PPM
1 week later	2	116.4561856	1.9999935	6.5 PPM

## Data Availability

Data have been uploaded to Figshare: https://doi.org/10.6084/m9.figshare.25210148.
